# Qubit Condensation for Assessing Efficacy of Molecular Simulation on Quantum Computers

**DOI:** 10.1021/acs.jpca.3c02583

**Published:** 2023-07-13

**Authors:** LeeAnn M. Sager-Smith, Scott E. Smart, David A. Mazziotti

**Affiliations:** † Department of Chemistry and The James Franck Institute, The University of Chicago, Chicago, Illinois 60637 United States; ‡ Department of Chemistry and Biochemistry, The University of California, Los Angeles, California 90095 United States

## Abstract

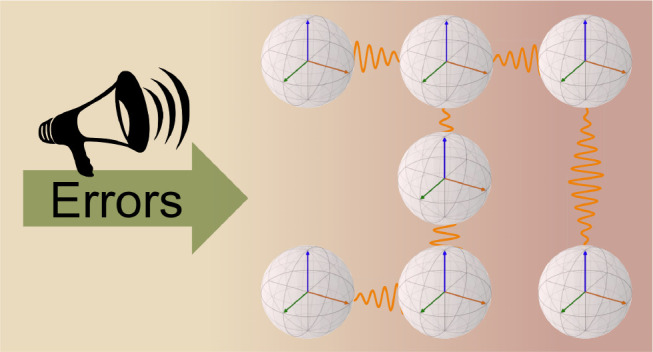

Quantum computers may demonstrate significant advantages over classical devices, as they are able to exploit a purely quantum-mechanical phenomenon known as entanglement in which a single quantum state simultaneously populates two-or-more classical configurations. However, due to environmental noise and device errors, elaborate quantum entanglement can be difficult to prepare on modern quantum computers. In this paper, we introduce a metric based on the condensation of qubits to assess the ability of a quantum device to simulate many-electron systems. Qubit condensation occurs when the qubits on a quantum computer condense into a single, highly correlated particle-hole state. While conventional metrics like gate errors and quantum volume do not directly target entanglement, the qubit-condensation metric measures the quantum computer’s ability to generate an entangled state that achieves nonclassical long-range order across the device. To demonstrate, we prepare qubit condensations on various quantum devices and probe the degree to which qubit condensation is realized via postmeasurement analysis. We show that the predicted ranking of the quantum devices is consistent with the errors obtained from molecular simulations of H_2_ using a contracted quantum eigensolver.

## Introduction

Quantum devices have recently emerged as potentially powerful tools for the demonstration of system-wide entanglement and long-range order,^1−10^ a task that can be difficult or expensive in classic computations. With an ability to simulate large degrees of quantum entanglement—important for the modeling of many chemical processes including those involving transition-metal complexes, energetic degeneracies, solid-state materials, and other systems^11−13^—quantum devices with quantum chemical algorithms are expected to compete with classical computers and methodologies for chemical computations.^14−17^

**Figure 1:**
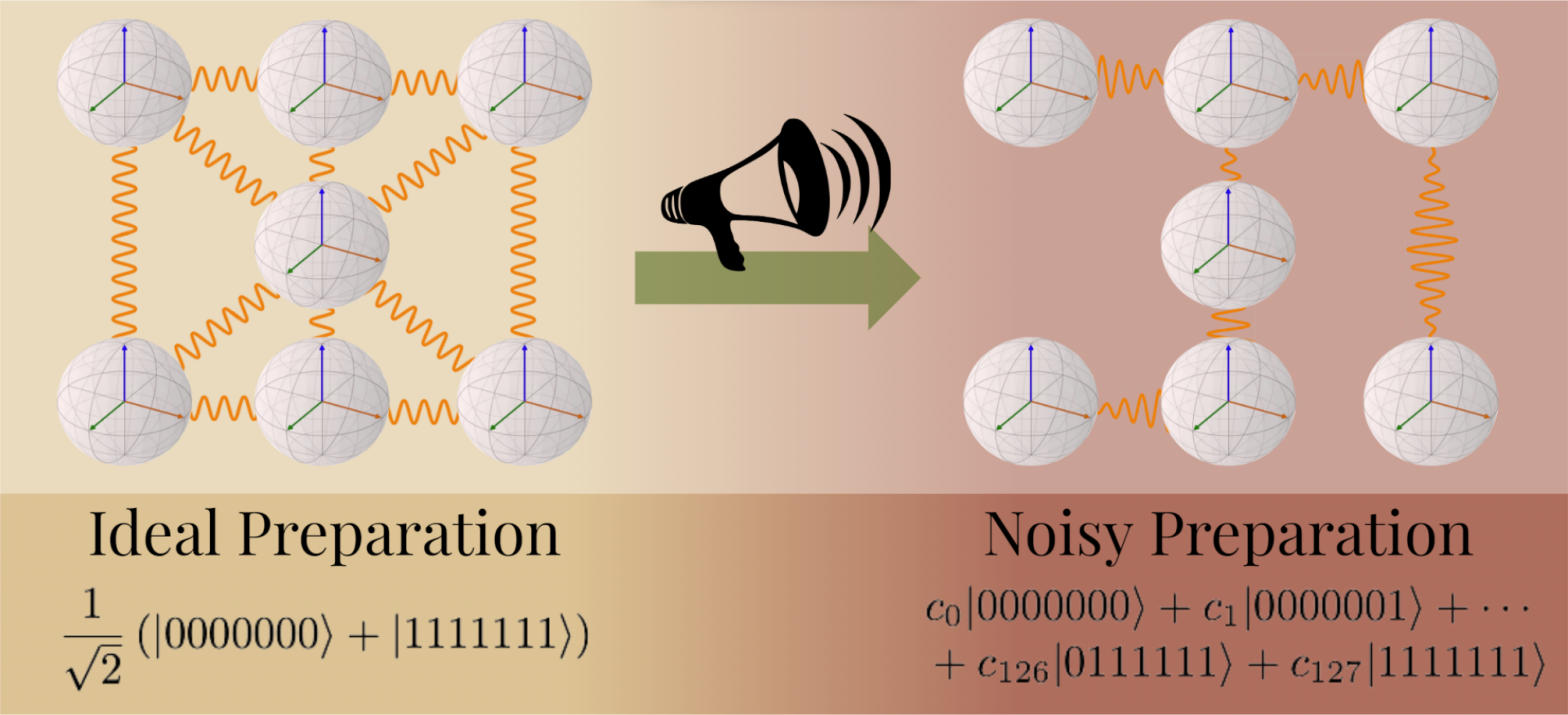
A schematic demonstrating noise in a NISQ device disrupting the correlations between a system of seven qubits prepared in the maximally entangled GHZ state. Each of the seven qubits is represented by a Bloch sphere with the correlation between each qubit depicted by the orange waves connecting them, with these correlations being disturbed going from an ideal noise-free and error-free quantum state preparation (left) to a noisy quantum state preparation (right).

However, algorithms for the accurate prediction of many-electron molecular energies and properties rely upon the ability of near-term intermediate-scale quantum (NISQ) devices to accurately accomplish state preparations and measurements,^6,18^ a requirement whose successful implementation can vary dramatically from device to device. Current NISQ computers are prone to experiencing environmental noise and device errors that disrupt long-range order (see Figure 1), often resulting in fragmented islands of correlated qubits instead of system-wide correlation,^8^ which can make simulating molecular systems difficult.

In this work, we introduce a novel metric for assessing the ability of quantum computers for modeling many-electron molecular systems. By preparing a maximally correlated qubit condensate state—a state where qubits on a quantum device condense into a single, highly correlated, particle-hole state—we directly probe the extent to which a given quantum device can achieve system-wide entanglement and long-range order. Because the modeling of quantum entanglement is what ultimately may separate quantum computers from classical computers, this ability provides a metric for the efficacy of various NISQ devices for the preparation of many-electron quantum systems that may be challenging for classical computers.

Currently, either component-level qubit and gate errors^19−21^ or system-level metrics such as quantum volume (QV)^22,23^ are seen as the conventional means of comparing various quantum devices against each other. While component-level metrics are useful while building quantum systems, they often fail to capture the behavior and errors of large quantum circuits on a given device.^24−26^ Thus, a system-level measure such as our metric or the QV is desirable. Unlike quantum volume, however, our qubit condensation metric is a specific measure of how well a quantum device can prepare a highly correlated state, making it a better predictive tool for comparing quantum devices for molecular simulations. Additionally, qubit condensation complements other measures of correlation such as mutual information.^27^

To test qubit condensation as a metric for many-electron quantum simulations, we compute the molecular energy of dihydrogen without error mitigation on several quantum devices using a contracted quantum eigensolver. The predicted ranking of the quantum devices from our qubit condensation analysis—which differs from the order of quantum volumes—is consistent with the errors obtained from the molecular simulation of H_2_. Our qubit condensate metric thus directly allows us to compare the accuracy with which NISQ devices are expected to treat many-electron quantum systems, which may aide researchers in the selection of the appropriate quantum device for quantum chemistry applications. Moreover, our metric may provide a measure along which future devices can be optimized in order to improve their ability to demonstrate quantum long-range order.

## Methods

A measure of the maximal quantum long-range order for the GHZ states that we prepare in this study is the signature of the condensation of particle-hole pairs as the maximal entanglement of the GHZ state corresponds to the maximal entanglement of particle-hole pairs.^8^ As such, we first detail the signature of such a qubit condensation, which will be used as a measure of the correlation for the quantum states prepared in this study. Further, the quantum solver we utilize to determine uncorrected molecular energies is the quantum anti-Hermitian contracted Schrödinger equation (QACSE) solver; as such, we additionally provide details pertaining to the QACSE.

### Signature of Qubit Condensation

Bose-Einstein condensation occurs when—at sufficiently low temperatures—multiple bosons all condense into a single quantum state^28,29^ and results in superfluidity—i.e., the frictionless flow of the constituent bosons.^30,31^ A computational signature of this type of condensation phenomena is a large eigenvalue in the one-boson reduced density matrix^32^ given by
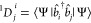
1where *b̂*_*i*_^†^ and *b̂*_*i*_ correspond to creation and annihilation operators for the *i*th bosonic orbital and where |Ψ⟩ is the full *N*-boson wave function in a finite basis set. This large eigenvalue corresponds to the largest orbital occupation of a given quantum state such that any eigenvalue above one indicates the beginnings of condensation behavior

As briefly described above, the maximal entanglement of the GHZ states we prepare in this study corresponds to the maximal degree of particle-hole condensation when we define each qubit to be a two-orbital system composed of a lower- and a higher-energy level corresponding to the |0⟩ and |1⟩ states, respectively. The particles and holes are fermions and thus must obey the Pauli exclusion principle such that they are unable to condense into a single orbital.^33^ However, particle-hole pairs are quasi-bosonic and hence can condense into a single particle-hole quantum state which we call a qubit condensate.^34,35^

Similar to the signature of a bosonic condensate being a large eigenvalue of a one-boson RDM, the computational signature of a particle-hole qubit condensate—denoted as *λ*_*G*_—is a large eigenvalue of the modified particle-hole reduced density matrix given by^36−38^
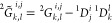
2where
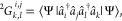
3is the unmodified particle-hole RDM, *â*_*i*_^†^ and *â*_*i*_ correspond to fermionic creation and annihilation operators for the *i*th fermionic orbital, ^1^*D*_*j*_^*i*^ is an element of the one-fermion RDM corresponding to indices *i* and *j*, and |Ψ⟩ is the full *N*-fermion wave function in a finite basis set. Note that modification to the particle-hole RDM (^2^*G*) is done in order to remove an extraneous large eigenvalue corresponding to the ground state to ground state transition. Explicitly, a large eigenvalue in the modified particle-hole matrix is a manifestation of the long-range order in this matrix and is a measure of entanglement.

For an *N*-fermion—and hence *N*-qubit—system, the largest possible signature of condensation is given by
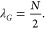
4

The GHZ state is expected to demonstrate this maximal degree of condensation on an ideal quantum device,^8^ and any deviation from this behavior would be due to errors on a given quantum device on which the state is prepared.

### Quantum Solver of the Anti-Hermitian Contracted Schrödinger Equation

Recently, Smart and Mazziotti^39^ have introduced a novel family of quantum eigensolvers, known as contracted quantum eigensolvers (CQE), that optimizes the lowest energy eigenvalue by solving the contracted Schrödinger equation (CSE)—which corresponds to the projection of the Schrödinger equation onto two-particle transitions from the wave function and is given by^40−46^
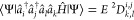
5where ^2^*D* is the two-particle reduced density matrices, *â*_*i*_^†^ and *â*_*i*_ are, again, fermionic creation and annihilation operators corresponding to the *i*^*th*^ orbital, |Ψ⟩ is the *N*-electron wave function, and *Ĥ* is the system Hamiltonian operator. Here we focus on the CQE that utilizes the anti-Hermitian part of the CSE—termed the anti-Hermitian CSE or ACSE and is given by^47−54^
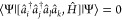
6—which depends upon both the 2-RDM and 3-RDM and has been utilized to solve for energies and properties of ground- and excited-state many-electron systems.^55−62^ The solution of the ACSE is closely related to the variational minimization of energy with respect to a series of two-body unitary transformations.^47,48,50^ In fact, the gradient of energy for the two-body unitary transformations is equivalent to the residual of the ACSE, which implies that the residual of the ACSE vanishes if and only if the energy gradient vanishes.^48^ As such, the ACSE can be utilized to iteratively apply a product of two-body unitary transformations on a reference wave function, which defines the quantum ACSE algorithm presented in refs (39 and 55). Specifically, in this framework, the density matrix of the (*n* + 1)th iteration (^2^*D*_*n*__+1_) is given by
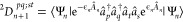
7where |Ψ_*n*_⟩ is the wave function that corresponds to the *n*th iteration with the initial wave function corresponding to the Hartree–Fock state |Ψ_0_⟩, where *ϵ*_*n*_ is an infinitesimal step, and where *Â*_*n*_ is an anti-Hermitian operator that can be set to the residual of the ACSE^39,50^ from eq 6. In our implementation of the QACSE, we generate all 2-RDMs on the quantum computer and compute *Â*_*n*_ by classically reconstructing the 3-RDM by its cumulant expansion^47,63^ with *O*(*r*^6^) cost where *r* is the rank of the one-electron basis set. A potentially more-efficient manner for the direct computation of *Â*_*n*_ on a quantum device has been introduced;^39^ however, this approach is not utilized for this study.

Note that while many error mitigation techniques have been utilized for the QACSE approach—such as those presented in ref (55)—as this study proposes an approach for comparing NISQ quantum hardware’s current utility for computation of many-electron systems rather than aiming to determine absolute energies of such systems, error mitigation techniques are not performed—allowing for the more direct comparison of each device against all others.

## Results

**Figure 2:**
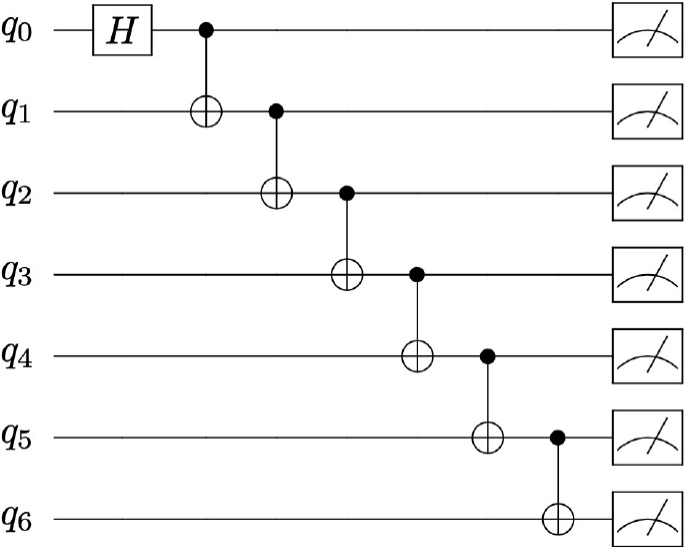
A schematic demonstrating the quantum state preparation that yields the seven-qubit GHZ state described by eq 8 with *N* = 7— where *H* represents the Hadamard gate and where two-qubit CNOT gates are represented such that the control qubit is specified by a dot connected to a target qubit represented by ⊕.

To demonstrate the accuracy with which specific quantum computers can construct highly correlated quantum states, we first prepare the “maximally-entangled”^64^*N*-qubit GHZ states (which are equivalently referred to as the Schrödinger’s Cat states) described by
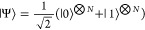
8—where |*i*⟩^⊗*N*^ represents the tensor product of the state |*i*⟩ for qubits *q*_0_ through *q*[*N* – 1]—on several of IBM’s seven-qubit quantum devices. The quantum state preparation for a seven-qubit GHZ state is shown in Figure 2, and details on this quantum state preparation are presented in the Supporting Information.

As demonstrated in ref (8), a characteristic of the GHZ state is the maximal entanglement of particle-hole pairs when each qubit is interpreted as a site consisting of one particle and two orbitals. Hence, an *N*-qubit GHZ state should demonstrate qubit condensation, namely, a large eigenvalue of the modified particle-hole reduced density matrix (^2^*G̃*) given by *N*/2 where *N* is the number of qubits and hence the number of particles. Any deviation from this expected value on a real quantum device, then, must be the result of errors in preparing and measuring the GHZ state on a given system. Therefore, measurement of the signature of qubit condensation for an *N*-qubit GHZ state on a given quantum device can serve as a measurement of the accuracy of that quantum device for preparing a highly correlated quantum state—the types of quantum states that will be required when utilizing quantum devices to compute energies and properties of highly correlated molecular systems.

**Figure 3:**
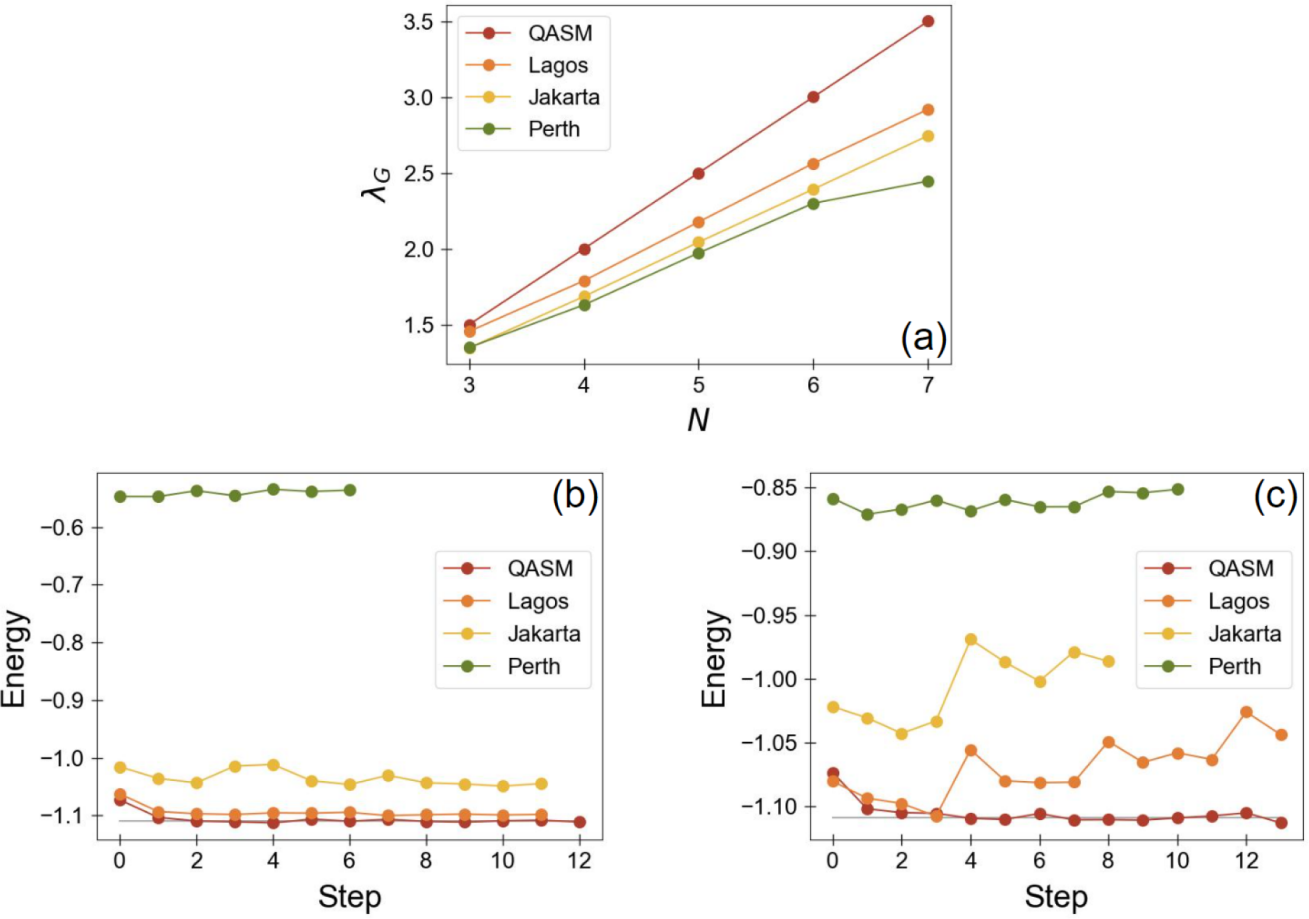
(a) The graph of the signature of qubit condensation (*λ*_*G*_) for an *N*-qubit GHZ state versus the number of qubits (*N*) for a QASM simulator (red), ibm_lagos (orange), ibmq_jakarta (yellow), and ibm_perth (green). The associated slopes as well as the *λ*_*G*_ corresponding to the three qubit (i.e., *N* = 3) GHZ state for each device in this plot are specified in Table 1. (b, c) For H_2_ with an internuclear distance of 1 Å, the energy at each iteration in the solution of the ACSE utilizing the STO-6G basis on a QASM simulator (red), ibm_lagos (orange), ibmq_jakarta (yellow), and ibm_perth (green) are shown for (b) the one-qubit and (c) the two-qubit calculations where the gray line is the expected FCI energy.

To this end, we prepare GHZ states composed of three to seven qubits on several of IBM’s seven-qubit quantum devices—specifically, ibm_lagos (QV = 32), ibm_perth (QV = 32), and ibmq_jakarta (QV = 16). As can be seen in Figure 3a, the graph of the signature of qubit condensation (*λ*_*G*_) versus the number of qubits (*N*) for an ideal quantum device—such as IBM’s QASM simulator that models a “perfect” quantum computer using classically computed probabilities—should be a line nearly exactly described by eq 4 (i.e., with slope *m* = 0.5) with any deviation resulting from sampling errors that approach zero as the number of samples (or “shots”) is increased.

The real devices, however, do not exhibit such ideal behavior. While the plots of *λ*_*G*_ vs *N* for real systems still appear linear, their slopes deviate from the expected value of 0.5 with this deviation demonstrating an overall decrease in the signature in qubit condensation for larger numbers of qubits. The signature of qubit condensation for a three-qubit subsystem as well as the slope associated with each real quantum system are shown in Table 1. The *λ*_*G*_ value for the three-qubit subsystem—with three being the smallest number of qubits capable of demonstrating condensation behavior (i.e., *λ*_*G*_ > 1)—indicates that all three real-world quantum devices are capable of supporting qubit condensation—a highly correlated phenomena—at small numbers of qubits, although the difference in the specific values does signify that even at this small number of qubits certain devices demonstrate more noise than others. Specifically, Lagos best matches the expected value of ^3^/_2_ = 1.5 with Jakarta and Perth showing much more notable deviations. On the other hand, this metric, specific to the three-qubit subsystem, does not sufficiently differentiate Jakarta and Perth, and the relative values between devices may not accurately reflect errors across the full seven-qubit quantum computers.

**Table 1:** Summary of the Slope of *λ*_*G*_ vs *N* for an *N*-Qubit GHZ State on a Simulator and Three Experimental Devices as well as the *λ*_*G*_ Value for a Three-Qubit GHZ State on Each Device, the Slope of Shannon Entropy versus *N* for an *N*-Qubit GHZ State on Each Device, and the Quantum Volume of Each Device

device	*m*(λ_G_)	λ_G_^(*N* = 3)^	*M*(S_e_)	QV
QASM simulator	0.500	1.500	0.000	—
Lagos	0.370	1.455	0.357	32
Jakarta	0.351	1.346	0.405	16
Perth	0.286	1.349	0.527	32

However, the slope of the lines for the *λ*_*G*_ vs *N* plots for each device gives a metric for how the degree of the highly correlated qubit condensation phenomena in the *N*-qubit GHZ state scales as *N* increases—with a slope approaching the ideal value of 0.5 indicating excellent preparation of larger, highly entangled states and slopes significantly diminished from 0.5 indicating large degrees of device error for the preparation of correlated states. As this metric corresponds to the preservation of correlation, we propose to utilize it to diagnose the relative efficacy of NISQ devices for the many-electron quantum calculations that heavily depend upon the accurate modeling of high degrees of correlation. As can be seen from Table 1, using this metric, one would predict that Lagos (with *m* = 0.370) would support the most-accurate quantum chemical calculations, followed by Jakarta (*m* = 0.351) with Perth (*m* = 0.286) being the least accurate by a significant margin.

To verify these predictions for the relative ability of different quantum devices to support the accurate computation of energies of many-electron quantum systems, we compare the non-error-mitigated calculation of the energy of a multielectron quantum system across all devices. Explicitly, we utilize the QACSE solver introduced in ref (39) to compute the ground-state energy of dihydrogen (H_2_) with an internuclear distance of 1 Angstrom and utilizing the minimal Slater-type orbital (STO-6G) basis on each quantum system of interest. Note that on the quantum computer, the dihydrogen molecule is represented in the QACSE algorithm by a two-qubit compact mapping that can be compressed to a one-qubit mapping through the application of appropriate tapering (as described in ref (55)).

As shown in Figure 3b,c, both one-qubit and two-qubit mappings are utilized to compute the energy of dihydrogen on each of the three seven-qubit quantum devices where Figure 3b,c displays the energy at each iteration in the solution of the QACSE. No attempts are made at error mitigation as we are not interested in accurately determining the energy of dihydrogen but rather we are interested in using the deviations from the expected energy to confirm the our predictions of which NISQ devices would be better for accurate calculations of molecular systems. For both the one-qubit (1Q, Figure 3b) and two-qubit (2Q, Figure 3c)—where QASM is a simulator that demonstrates ideal behavior and where the gray line represents the exact FCI energy for H_2_—it is clear that Lagos supports the most-accurate computation of H_2_’s energy followed by Jakarta with Perth being a distant third—which is exactly what we predict with our proposed metric.

Finally, we compare our metric for determination of appropriate NISQ devices for the computation of many-electron chemical systems against two previously established metrics for determination of the capabilities of quantum systems. The first metric, quantum volume,^22,23^ was introduced by IBM in ref (23) to compare the capabilities of NISQ devices, and the quantum volumes for the devices employed in this study are provided in Table 1. The quantum volume gives a quantitative measure to the largest random circuit of equal width and depth that the computer successfully implements such that systems with high fidelity, high connectivity, and a high number of possible gates have higher quantum volumes. However, many devices that may behave in vastly different ways have the same quantum volume—making this metric only somewhat useful for differentiating between devices. Further, it is not clear that QV directly applies to the ability of a given device to support high degrees of correlation, as needed for molecular simulation. In fact, in this instance, using quantum volume as a metric one would predict Perth to be better-able to compute the energy of dihydrogen relative to Jakarta, which is not consistent with the results in Figure 3b,c. Hence, our metric is better able to predict the behavior of NISQ devices in terms of viability for many-electron calculations than quantum volume.

**Figure 4:**
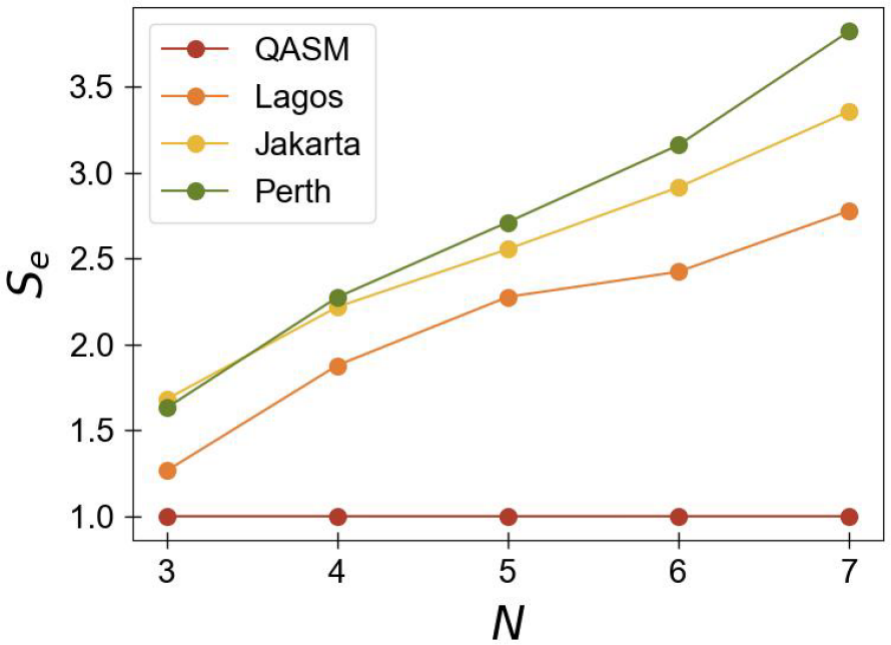
Graph of Shannon entropy (*S*_*e*_) for an *N*-qubit GHZ state versus the number of qubits (*N*) for a QASM simulator (red), ibm_lagos (orange), ibmq_jakarta (yellow), and ibm_perth (green). The associated slopes for each device are shown in Table 1.

The second metric of interest is the one based on the slope of the plot of Shannon entropy versus number of qubits for *N*-qubit GHZ states proposed by Hunt et al. in ref (65). As can be seen in Figure 4, we construct a figure of Shannon entropy (*S*_*e*_) versus number of qubits (*N*) for each quantum device employed in this study. (Specifics regarding the calculation of Shannon entropy are included in the Supporting Information.) We then obtain the slopes of these plots, which are given in Table 1 with the extent to which slopes deviate from the expected value of zero being a possible metric for a quantum computer’s error. Using this criteria, then, one would expect Lagos to be the most accurate quantum system, followed by Jakarta and then Perth—which agrees with both the QACSE results and additionally the metric we propose. While our qualitative predictions agree with those from Hunt’s metric, the Shannon entropy is derived from the diagonal elements of the *N*-qubit density matrix and hence does not directly probe the correlation between pairs of qubits. As the measurement of correlation is essential for the accurate computation of many-electron quantum systems, our metric—the slope of *λ*_*G*_ vs *N*—which does directly probe two-body correlation through probing the modified particle-hole density matrix may be better suited for differentiating between NISQ devices for quantum-chemical simulations.

## Discussion and Conclusions

Here we introduce a novel metric for benchmarking near-term intermediate-scale quantum devices focused on the ability of such quantum systems to reliably prepare and probe a maximally entangled quantum system. As quantum chemical systems of interest often display high degrees of quantum long-range order, such a benchmark is taken to be predictive of the efficacy with which a given quantum device can simulate a many-electron atom or molecule. Specifically, we utilize the signature of the maximally entangled GHZ state—i.e., the largest eigenvalue of the modified particle-hole RDM (*λ*_*G*_) that should ideally approach *N*/2 for a *N*-qubit GHZ state. This signature—which is related to other measures of correlation including mutual information^27^—can either be utilized as a singular metric that can quantify the “loss” of correlation for a given subsystem of qubits, or—as we have proposed—one can construct a plot of *λ*_*G*_ versus *N* for a given device in order to determine the slope of the resultant linear fit. This slope of *λ*_*G*_ versus *N* is then a metric that describes the ability of an entire quantum system to maintain quantum long-range order—the closer to 1/2, the more accurately the quantum device is capable of preparing and measuring highly correlated systems.

Using this metric, we then benchmark three of IBM’s NISQ quantum devices, and from this analysis, we predict Lagos to be best-suited for calculating molecular energies and properties followed by Jakarta and then Perth. By directly computing the energy of dihyrogen using the QACSE solver outlined in ref (39), we verify this prediction by identifying that the dihydrogen energy is best computed by Lagos followed by Jakarta with Perth being the least accurate—just as our metric forecasts. Of note is that if one were to utilize the IBM-provided quantum volume as the metric by which such a prediction were made, Lagos and Perth would be expected to demonstrate roughly equivalent accuracy for computing chemical properties with Jakarta—having the lowest quantum volume—being the least accurate.

Comparing to the more established benchmark of quantum volume, our proposed metric not only allows for more-granular discernment in comparing devices with the same quantum volume (as the slope values are less likely to be identical) but additionally overcomes a shortcoming of quantum volume—namely, that quantum volume does not necessarily directly probe metrics related to the preparation of molecular systems which may limit its applicability to prescribing which quantum computers are “best” for such applications. By directly corresponding to a measurement of entanglement, the slope of *λ*_*G*_ versus *N* allows us to correctly identify that, although Perth has a higher quantum volume than Jakarta, Jakarta is the better system for modeling molecular systems.

One aspiration of quantum computing since the days of Feynman^66^ has been the achievement of quantum advantage over traditional classical computing for the simulation of atoms and molecules. As the construction of the wave function on a classical system scales exponentially with the number of orbital-based configurations and—in principle—quantum computers offer the possibility of nonexponential scaling, such an advantage may be obtained as quantum algorithms and *especially* hardware mature. In order to obtain quantum advantage, however, highly correlated molecular systems need to be modeled on real-world devices in an accurate manner—a feat that is difficult on modern NISQ devices for complex state preparations and more than a few qubits. The benchmark that we propose—in addition to allowing for the comparison of current quantum systems—may act as a metric along which future devices can be improved in order to better demonstrate quantum long-range order and hence may serve as an aide in the search for quantum advantage in molecular simulations.

## Supporting Information Available

Relevant details on the quantum algorithm utilized to prepare the *N*-qubit GHZ states presented in the article, the quantum tomography of the particle-hole reduced density matrix, the determination of average Shannon entropies, the quantum anti-Hermitian Schrödinger equation, and the experimental quantum devices employed (PDF)

## References

[ref1] Verstraete F.; Cirac J. I.; Latorre J. I. Quantum circuits for strongly correlated quantum systems. Physical Review A 2009, 79, 032316 10.1103/PhysRevA.79.032316.

[ref2] Smith A.; Kim M. S.; Pollmann F.; Knolle J. Simulating quantum many-body dynamics on a current digital quantum computer. NPJ Quantum Information 2019, 5, 106 10.1038/s41534-019-0217-0.

[ref3] Mooney G. J.; Hill C. D.; Hollenberg L. C. L. Entanglement in a 20-Qubit Superconducting Quantum Computer. Scientific Reports 2019, 9, 13465 10.1038/s41598-019-49805-7. 31530848 PMC6748943

[ref4] Huang H.-L.; Wu D.; Fan D.; Zhu X. Superconducting quantum computing: a review. Science China Information Sciences 2020, 63, 180501 10.1007/s11432-020-2881-9.

[ref5] Mcardle S.; Endo S.; Aspuru-Guzik A.; Benjamin S. C.; Yuan X. Quantum computational chemistry. Rev. Mod. Phys. 2020, 92, 015003 10.1103/RevModPhys.92.015003.

[ref6] Head-Marsden K.; Flick J.; Ciccarino C. J.; Narang P. Quantum information and algorithms for correlated quantum matter. Chem. Rev. 2021, 121, 3061–3120. 10.1021/acs.chemrev.0c00620. 33326218

[ref7] Smart S. E.; Mazziotti D. A. Quantum-classical hybrid algorithm using an error-mitigating N -representability condition to compute the Mott metal-insulator transition. Phys. Rev. A (Coll. Park.) 2019, 100, 022517 10.1103/PhysRevA.100.022517.

[ref8] Sager L. M.; Smart S. E.; Mazziotti D. A. Preparation of an Exciton Condensate of Photons on a 53-Qubit Quantum Computer. Phys. Rev. Research 2020, 2, 043205 10.1103/PhysRevResearch.2.043205.

[ref9] Sager L. M.; Mazziotti D. A. Cooper-pair condensates with nonclassical long-range order on quantum devices. Phys. Rev. Res. 2022, 4, 013003 10.1103/PhysRevResearch.4.013003.

[ref10] Sager L. M.; Mazziotti D. A. Entangled phase of simultaneous fermion and exciton condensations realized. Phys. Rev. B 2022, 105, L121105 10.1103/PhysRevB.105.035143.

[ref11] Helgaker T.; Jorgensen P.; Olsen J. Molecular electronic-structure theory; John Wiley & Sons: Nashville, TN, 2013.

[ref12] Lischka H.; Nachtigallová D.; Aquino A. J. A.; Szalay P. G.; Plasser F.; Machado F. B. C.; Barbatti M. Multireference approaches for excited states of molecules. Chem. Rev. 2018, 118, 7293–7361. 10.1021/acs.chemrev.8b00244. 30040389

[ref13] Evangelista F. A. Perspective: Multireference coupled cluster theories of dynamical electron correlation. J. Chem. Phys. 2018, 149, 030901 10.1063/1.5039496. 30037266

[ref14] Aspuru-Guzik A.; Dutoi A. D.; Love P. J.; Head-Gordon M. Simulated quantum computation of molecular energies. Science 2005, 309, 1704–1707. 10.1126/science.1113479. 16151006

[ref15] Lloyd S. Universal quantum simulators. Science 1996, 273, 1073–1078. 10.1126/science.273.5278.1073. 8688088

[ref16] Lu D.; Xu B.; Xu N.; Li Z.; Chen H.; Peng X.; Xu R.; Du J. Quantum chemistry simulation on quantum computers: theories and experiments. Phys. Chem. Chem. Phys. 2012, 14, 9411–9420. 10.1039/c2cp23700h. 22652702

[ref17] Elfving V. E.; Broer B. W.; Webber M.; Gavartin J.; Halls M. D.; Lorton K. P.; Bochevarov A. How will quantum computers provide an industrially relevant computational advantage in quantum chemistry? arXiv 2020, 1–20; https://arxiv.org/abs/2009.12472. Date Accessed: 2023.06.12

[ref18] Preskill J. Quantum Computing in the NISQ era and beyond. Quantum 2018, 2, 79 10.22331/q-2018-08-06-79.

[ref19] Chuang I. L.; Nielsen M. A. Prescription for experimental determination of the dynamics of a quantum black box. J. Mod. Opt. 1997, 44, 2455–2467. 10.1080/09500349708231894.

[ref20] Knill E.; Leibfried D.; Reichle R.; Britton J.; Blakestad R. B.; Jost J. D.; Langer C.; Ozeri R.; Seidelin S.; Wineland D. J. Randomized benchmarking of quantum gates. Phys. Rev. A 2008, 77, 012307 10.1103/PhysRevA.77.012307.

[ref21] Magesan E.; Gambetta J. M.; Emerson J. Characterizing quantum gates via randomized benchmarking. Physical Review A 2012, 85, 042311 10.1103/PhysRevA.85.042311.

[ref22] Baldwin C. H.; Mayer K.; Brown N. C.; Ryan-Anderson C.; Hayes D. Re-examining the quantum volume test: Ideal distributions, compiler optimizations, confidence intervals, and scalable resource estimations. Quantum 2022, 6, 707 10.22331/q-2022-05-09-707.

[ref23] Cross A. W.; Bishop L. S.; Sheldon S.; Nation P. D.; Gambetta J. M. Validating quantum computers using randomized model circuits. Phys. Rev. A 2019, 100, 032328 10.1103/PhysRevA.100.032328.

[ref24] Lubinski T.; Johri S.; Varosy P.; Coleman J.; Zhao L.; Necaise J.; Baldwin C. H.; Mayer K.; Proctor T. Application-Oriented Performance Benchmarks for Quantum Computing. IEEE Transactions on Quantum Engineering 2023, 4, 1–32. 10.1109/TQE.2023.3253761.

[ref25] Proctor T.; Rudinger K.; Young K.; Nielsen E.; Blume-Kohout R. Measuring the capabilities of quantum computers. Nat. Phys. 2022, 18, 75–79. 10.1038/s41567-021-01409-7.

[ref26] Sarovar M.; Proctor T.; Rudinger K.; Young K.; Nielsen E.; Blume-Kohout R. Detecting crosstalk errors in quantum information processors. Quantum 2020, 4, 321 10.22331/q-2020-09-11-321. PMC7588494 33106482

[ref27] Tkachenko N. V.; Sud J.; Zhang Y.; Tretiak S.; Anisimov P. M.; Arrasmith A. T.; Coles P. J.; Cincio L.; Dub P. A. Correlation-Informed Permutation of Qubits for Reducing Ansatz Depth in the Variational Quantum Eigensolver. PRX Quantum 2021, 2, 020337 10.1103/PRXQuantum.2.020337.

[ref28] Bose S. N.; Einstein A. Planck’s Law and Light Quantum Hypothesis. Zeitscrift für Physik 1924, 26, 178 10.1007/BF01327326.

[ref29] Einstein A. Quantentheorie des einatomigen idealen Gases. K.P.A.W. 1924, 261–267.

[ref30] London F. On Bose-Einstein condensation. Phys. Rev. 1938, 54, 947–954. 10.1103/PhysRev.54.947.

[ref31] Tisza L. The Theory of Liquid Helium. Phys. Rev. 1947, 72, 838–854. 10.1103/PhysRev.72.838.

[ref32] Penrose O.; Onsager L. Bose-Einstein condensation and liquid helium. Phys. Rev. 1956, 104, 576–584. 10.1103/PhysRev.104.576.

[ref33] Pauli W. The Connection Between Spin and Statistics. Phys. Rev. 1940, 58, 716–722. 10.1103/PhysRev.58.716.

[ref34] Fil D. V.; Shevchenko S. I. Electron-hole Superconductivity (Review). Low Temp. Phys. 2018, 44, 867–909. 10.1063/1.5052674.

[ref35] Keldysh L. V. Coherent states of excitons. Physics-Uspekhi 2017, 60, 1180–1186. 10.3367/UFNe.2017.10.038227.

[ref36] Safaei S.; Mazziotti D. A. Quantum signature of exciton condensation. Phys. Rev. B 2018, 98, 045122 10.1103/PhysRevB.98.045122.

[ref37] Garrod C.; Rosina M. Particle-Hole Matrix: Its Connection with the Symmetries and Collective Features of the Ground State. J. Math. Phys. 1969, 10, 1855–1861. 10.1063/1.1664770.

[ref38] Kohn W.; Sherrington D. Two Kinds of Bosons and Bose Condensates. Rev. Mod. Phys. 1970, 42, 1–11. 10.1103/RevModPhys.42.1.

[ref39] Smart S. E.; Mazziotti D. A. Quantum solver of contracted eigenvalue equations for scalable molecular simulations on quantum computing devices. Phys. Rev. Lett. 2021, 126, 070504 10.1103/PhysRevLett.126.070504. 33666467

[ref40] Mazziotti D. A. Contracted Schrödinger equation: determining quantum energies and two-particle density matrices without wave functions. Phys. Rev. A 1998, 57, 4219–4234. 10.1103/PhysRevA.57.4219.

[ref41] Nakatsuji H.; Yasuda K. Direct Determination of the Quantum-Mechanical Density Matrix Using the Density Equation. Phys. Rev. Lett. 1996, 76, 1039–1042. 10.1103/PhysRevLett.76.1039. 10061618

[ref42] Yasuda K.; Nakatsuji H. Direct determination of the quantum-mechanical density matrix using the density equation. II. Phys. Rev. A 1997, 56, 2648–2657. 10.1103/PhysRevA.56.2648. 10061618

[ref43] Colmenero F.; Valdemoro C. Approximating q-order reduced density matrices in terms of the lower-order ones. II. Applications. Phys. Rev. A 1993, 47, 979–985. 10.1103/PhysRevA.47.979. 9909018

[ref44] Valdemoro C.; Tel L. M.; Pérez-Romero E.; Alcoba D. R. Four new forms of the contracted Schrödinger equation and their connection with the second-order hypervirial condition. Int. J. Quantum Chem. 2008, 108, 1090–1096. 10.1002/qua.21576.

[ref45] Mazziotti D. A. Variational method for solving the contracted Schrödinger equation through a projection of the N-particle power method onto the two-particle space. J. Chem. Phys. 2002, 116, 1239–1249. 10.1063/1.1430257.

[ref46] Mazziotti D. A. Comparison of contracted Schrödinger and coupled-cluster theories. Phys. Rev. A 1999, 60, 4396–4408. 10.1103/PhysRevA.60.4396.

[ref47] Mazziotti D. A. Anti-Hermitian Contracted Schrödinger Equation: Direct Determination of the Two-Electron Reduced Density Matrices of Many-Electron Molecules. Phys. Rev. Lett. 2006, 97, 143002 10.1103/PhysRevLett.97.143002. 17155245

[ref48] Mazziotti D. A. Multireference many-electron correlation energies from two-electron reduced density matrices computed by solving the anti-Hermitian contracted Schrödinger equation. Phys. Rev. A 2007, 76, 052502 10.1103/PhysRevA.76.052502.

[ref49] Mazziotti D. A. Two-electron reduced density matrices from the anti-Hermitian contracted Schrödinger equation: Enhanced energies and properties with larger basis sets. J. Chem. Phys. 2007, 126, 184101 10.1063/1.2723115. 17508786

[ref50] Mazziotti D. A. Anti-Hermitian part of the contracted Schrödinger equation for the direct calculation of two-electron reduced density matrices. Phys. Rev. A 2007, 75, 022505 10.1103/PhysRevA.75.022505. 17155245

[ref51] Rothman A. E.; Foley J. J.; Mazziotti D. A. Open-shell energies and two-electron reduced density matrices from the anti-Hermitian contracted Schrödinger equation: A spin-coupled approach. Phys. Rev. A 2009, 80, 052508 10.1103/PhysRevA.80.052508.

[ref52] Gidofalvi G.; Mazziotti D. A. Direct calculation of excited-state electronic energies and two-electron reduced density matrices from the anti-Hermitian contracted Schrödinger equation. Phys. Rev. A 2009, 80, 022507 10.1103/PhysRevA.80.022507.

[ref53] Sand A. M.; Mazziotti D. A. Enhanced computational efficiency in the direct determination of the two-electron reduced density matrix from the anti-Hermitian contracted Schrödinger equation with application to ground and excited states of conjugated *π*-systems. J. Chem. Phys. 2015, 143, 134110 10.1063/1.4931471. 26450295

[ref54] Mazziotti D. A. Reduced-Density-Matrix Mechanics: With Application to Many-Electron Atoms and Molecule. Adv. Chem. Phys. 2007, 134, 19 10.1002/9780470106600.ch3.

[ref55] Smart S. E.; Boyn J.-N.; Mazziotti D. A. Resolving correlated states of benzyne with an error-mitigated contracted quantum eigensolver. Phys. Rev. A 2022, 105, 022405 10.1103/PhysRevA.105.022405.

[ref56] Smart S. E.; Scrape P. G.; Butler L. J.; Mazziotti D. A. Using reduced density matrix techniques to capture static and dynamic correlation in the energy landscape for the decomposition of the *CH*_2_*CH*_2_*ONO* radical and support a non-IRC pathway. J. Chem. Phys. 2018, 149, 024302 10.1063/1.5024512. 30007389

[ref57] Schlimgen A. W.; Mazziotti D. A. Static and dynamic electron correlation in the ligand noninnocent oxidation of nickel dithiolates. J. Phys. Chem. A 2017, 121, 9377–9384. 10.1021/acs.jpca.7b09567. 29155587

[ref58] Sturm E. J.; Mazziotti D. A. Highly accurate excited-state energies from direct computation of the 2-electron reduced density matrix by the anti-Hermitian contracted Schrödinger equation. Mol. Phys. 2016, 114, 335–343. 10.1080/00268976.2015.1074739.

[ref59] Snyder J. W. Jr.; Mazziotti D. A. Photoexcited tautomerization of vinyl alcohol to acetylaldehydevia a conical intersection from contracted Schrödinger theory. Phys. Chem. Chem. Phys. 2012, 14, 1660–1667. 10.1039/C2CP23065H. 22194059

[ref60] Snyder J. W.; Mazziotti D. A. Photoexcited conversion of gauche-1,3-butadiene to bicyclobutane via a conical intersection: Energies and reduced density matrices from the anti-Hermitian contracted Schrödinger equation. J. Chem. Phys. 2011, 135, 024107 10.1063/1.3606466. 21766925

[ref61] Greenman L.; Mazziotti D. A. Balancing single- and multi-reference correlation in the chemiluminescent reaction of dioxetanone using the anti-Hermitian contracted Schrödinger equation. J. Chem. Phys. 2011, 134, 174110 10.1063/1.3585691. 21548676

[ref62] Snyder J. W. Jr; Rothman A. E.; Foley J. J. 4th; Mazziotti D. A. Conical intersections in triplet excited states of methylene from the anti-Hermitian contracted Schrödinger equation. J. Chem. Phys. 2010, 132, 154109 10.1063/1.3394020. 20423170

[ref63] Mazziotti D. A. Approximate solution for electron correlation through the use of Schwinger probes. Chem. Phys. Lett. 1998, 289, 419–427. 10.1016/S0009-2614(98)00470-9.

[ref64] Walter M.; Gross D.; Eisert J. Multipartite Entanglement. Quantum Information 2016, 293–330. 10.1002/9783527805785.ch14.

[ref65] Jansen N. D.; Loucks M.; Gilbert S.; Fleming-Dittenber C.; Egbert J.; Hunt K. L. C. Shannon and von Neumann entropies of multi-qubit Schrödinger’s cat states. Phys. Chem. Chem. Phys. 2022, 24, 7666–7681. 10.1039/D1CP05255A. 35297927

[ref66] Feynman R. P. Simulating Physics with Computers. Int. J. Theor. Phys. 1982, 21, 467–488. 10.1007/BF02650179.

