# Supplementary material for: Qubit Condensation for Assessing Efficacy of Molecular Simulation on Quantum Computers
Source: J Phys Chem A. 2023 Jul 13;127(29):6032–9. doi: 10.1021/acs.jpca.3c02583 (PMC10388352; doi:10.1021/acs.jpca.3c02583)
Supplement: Supplementary file 1 — jp3c02583_si_001.pdf [file jp3c02583_si_001.pdf]

Supporting Information: Qubit Condensation for Assessing Efficacy of Molecular Simulation on Quantum Computers

LeeAnn M. Sager-Smith,[1] Scott E. Smart,[2] and David A. Mazziotti[1,*]

[1]*Department of Chemistry and The James Franck Institute, The University of Chicago, Chicago, IL 60637 USA*

[2]*Department of Chemistry and Biochemistry, The University of California, Los Angeles, CA 90095 USA*

We include relevant details on the quantum algorithm utilized to prepare the N-qubit GHZ states presented in this article; the quantum tomography of the particle-hole reduced density matrix; the determination of average Shannon entropies; the quantum anti-Hermitian Schrödinger equation; and the experimental quantum devices employed.

GHZ state preparation.

The GHZ state described in Eq. (8) is prepared according to

$$|\Psi\rangle = C_{N-2}^{N-1} \cdots C_2^3 C_1^2 H_1 |0\rangle^{\otimes N} \tag{1}$$

for an N-qubit state where H_i corresponds to a Hadamard gate applied to qubit i—which does the following mappings

$$|0\rangle \Rightarrow \frac{|0\rangle + |1\rangle}{\sqrt{2}} \tag{2}$$

and

$$|1\rangle \Rightarrow \frac{|0\rangle - |1\rangle}{\sqrt{2}} \tag{3}$$

—and where C_i^j is a controlled-NOT (CNOT) gate with control i and target j. The specific state preparation for a seven-qubit GHZ state according to this methodology is pictorially represented in Fig. 2. While many studies have centered on identifying the optimal state preparations for such GHZ states [1–4], the algorithm employed in this study [5] is chosen as it is easily-implemented and easily-generalizable to any number of qubits.

Quantum tomography of the particle-hole RDM.

The elements of the modified particle-hole reduced density matrix given by Eq. (2) are determined from a given quantum computation through their translation into the bases of Pauli matrices with each of the Pauli expectation values being directly probed on a quantum device.

First, let's focus on the one-particle reduced density matrix (${}^1D_i^j$) terms for the elements of ${}^2\tilde{G}$. Note that there are no non-zero two-qubit 1-RDM terms as the 1-RDM simplifies to a block diagonal form with respect to a single qubit. For a 1-RDM to be non-zero, then, it must be a one-qubit 1-RDM of the form

	$\hat{a}_{p,0}$	$\hat{a}_{p,1}$
$\hat{a}_{p,0}^\dagger$	$\hat{a}_{p,0}^\dagger \hat{a}_{p,0}$	$\hat{a}_{p,0}^\dagger \hat{a}_{p,1}$
$\hat{a}_{p,1}^\dagger$	$\hat{a}_{p,1}^\dagger \hat{a}_{p,0}$	$\hat{a}_{p,1}^\dagger \hat{a}_{p,1}$

(4)

where p is the index for a given qubit and $\hat{a}_p^\dagger$ and $\hat{a}_p$ are, respectively, creation and annihilation operators for qubit p. Each matrix element shown in Eq. (4) can be represented as a linear combination of Pauli matrices according to

$$\hat{a}_{p,0}^\dagger \hat{a}_{p,1} = \begin{pmatrix} 0 & 1 \\ 0 & 0 \end{pmatrix} = \frac{1}{2}\left(X_p + iY_p\right), \tag{5}$$

$$\hat{a}_{p,1}^\dagger \hat{a}_{p,0} = \begin{pmatrix} 0 & 0 \\ 1 & 0 \end{pmatrix} = \frac{1}{2}\left(X_p - iY_p\right), \tag{6}$$

$$\hat{a}_{p,0}^\dagger \hat{a}_{p,0} = \begin{pmatrix} 1 & 0 \\ 0 & 0 \end{pmatrix} = \frac{1}{2}\left(\hat{I} + Z_p\right), \tag{7}$$

and

$$\hat{a}_{p,1}^\dagger \hat{a}_{p,1} = \begin{pmatrix} 0 & 0 \\ 0 & 1 \end{pmatrix} = \frac{1}{2}\left(\hat{I} - Z_p\right). \tag{8}$$

where the expectation value of each matrix element for a given qubit p can then be obtained by directly obtaining the expectation values for X_p, Y_p, and Z_p for a given state preparation.

The particle-hole RDM (^2G matrix) can be represented as a $4N \times 4N$ matrix composed of N^2 4×4 sub-matrices represented by

$$\begin{array}{c|cccc}
 & \hat{a}_{q,0}^\dagger \hat{a}_{q,0} & \hat{a}_{q,1}^\dagger \hat{a}_{q,0} & \hat{a}_{q,0}^\dagger \hat{a}_{q,1} & \hat{a}_{q,1}^\dagger \hat{a}_{q,1} \\
\hline
\hat{a}_{p,0}^\dagger \hat{a}_{p,0} & \hat{a}_{p,0}^\dagger \hat{a}_{p,0}\hat{a}_{q,0}^\dagger \hat{a}_{q,0} & \hat{a}_{p,0}^\dagger \hat{a}_{p,0}\hat{a}_{q,1}^\dagger \hat{a}_{q,0} & \hat{a}_{p,0}^\dagger \hat{a}_{p,0}\hat{a}_{q,0}^\dagger \hat{a}_{q,1} & \hat{a}_{p,0}^\dagger \hat{a}_{p,0}\hat{a}_{q,1}^\dagger \hat{a}_{q,1} \\
\hat{a}_{p,0}^\dagger \hat{a}_{p,1} & \hat{a}_{p,0}^\dagger \hat{a}_{p,1}\hat{a}_{q,0}^\dagger \hat{a}_{q,0} & \hat{a}_{p,0}^\dagger \hat{a}_{p,1}\hat{a}_{q,1}^\dagger \hat{a}_{q,0} & \hat{a}_{p,0}^\dagger \hat{a}_{p,1}\hat{a}_{q,0}^\dagger \hat{a}_{q,1} & \hat{a}_{p,0}^\dagger \hat{a}_{p,1}\hat{a}_{q,1}^\dagger \hat{a}_{q,1} \\
\hat{a}_{p,1}^\dagger \hat{a}_{p,0} & \hat{a}_{p,1}^\dagger \hat{a}_{p,0}\hat{a}_{q,0}^\dagger \hat{a}_{q,0} & \hat{a}_{p,1}^\dagger \hat{a}_{p,0}\hat{a}_{q,1}^\dagger \hat{a}_{q,0} & \hat{a}_{p,1}^\dagger \hat{a}_{p,0}\hat{a}_{q,0}^\dagger \hat{a}_{q,1} & \hat{a}_{p,1}^\dagger \hat{a}_{p,0}\hat{a}_{q,1}^\dagger \hat{a}_{q,1} \\
\hat{a}_{p,1}^\dagger \hat{a}_{p,1} & \hat{a}_{p,1}^\dagger \hat{a}_{p,1}\hat{a}_{q,0}^\dagger \hat{a}_{q,0} & \hat{a}_{p,1}^\dagger \hat{a}_{p,1}\hat{a}_{q,1}^\dagger \hat{a}_{q,0} & \hat{a}_{p,1}^\dagger \hat{a}_{p,1}\hat{a}_{q,0}^\dagger \hat{a}_{q,1} & \hat{a}_{p,1}^\dagger \hat{a}_{p,1}\hat{a}_{q,1}^\dagger \hat{a}_{q,1}.
\end{array} \tag{9}$$

where each element can again be written as a linear combination of strings of Pauli matrices obtained as is shown for the matrix element $\hat{a}_{p,0}^\dagger \hat{a}_{p,1}\hat{a}_{q,1}^\dagger \hat{a}_{q,1}$:

$$\begin{aligned}
\hat{a}_{p,0}^\dagger \hat{a}_{p,1}\hat{a}_{q,1}^\dagger \hat{a}_{q,1} &= \left(\hat{a}_{p,0}^\dagger \hat{a}_{p,1}\right) \otimes \left(\hat{a}_{q,1}^\dagger \hat{a}_{q,1}\right) \\
&= \left[\frac{1}{2}\left(X_p + iY_p\right)\right] \otimes \left[\frac{1}{2}\left(\hat{I}_q - Z_q\right)\right] \\
&= \frac{1}{4}\left[X_p \otimes \hat{I}_q - X_p \otimes Z_q + iY_p \otimes \hat{I}_q - iY_p \otimes Z_q\right],
\end{aligned} \tag{10}$$

Note that $Y_p \otimes Z_q$ is one of nine possible two-qubit tensor products. The value of all matrix elements for ^2G can then be computed by directly probing the one-qubit and two-qubit Pauli expectations values on a

given quantum device and performing the appropriate linear combination such as the one shown in Eq. (10).

The modified particle-hole reduced density matrix (${}^2\tilde{G}$) is then obtained as a $4N \times 4N$ matrix composed of N^2 4×4 sub-matrices with these submatrices being given by those shown in Eq. (9) with the block modification below subtracted off in order to remove an extraneous ground-state-to-ground-state transition.

$$
\begin{array}{c|cccc}
 & \hat{a}^{\dagger}_{q,0}\hat{a}_{q,0} & \hat{a}^{\dagger}_{q,1}\hat{a}_{q,0} & \hat{a}^{\dagger}_{q,0}\hat{a}_{q,1} & \hat{a}^{\dagger}_{p,1}\hat{a}_{p,1} \\
\hline
\hat{a}^{\dagger}_{p,0}\hat{a}_{p,0} & {}^1D_p[0,0]{}^1D_q[0,0] & {}^1D_p[0,0]{}^1D_q[0,1] & {}^1D_p[0,0]{}^1D_q[1,0] & {}^1D_p[0,0]{}^1D_q[1,1] \\
\hat{a}^{\dagger}_{p,0}\hat{a}_{p,1} & {}^1D_p[0,1]{}^1D_q[0,0] & {}^1D_p[0,1]{}^1D_q[0,1] & {}^1D_p[0,1]{}^1D_q[1,0] & {}^1D_p[0,1]{}^1D_q[1,1] \\
\hat{a}^{\dagger}_{p,1}\hat{a}_{p,0} & {}^1D_p[1,0]{}^1D_q[0,0] & {}^1D_p[1,0]{}^1D_q[0,1] & {}^1D_p[1,0]{}^1D_q[1,0] & {}^1D_p[1,0]{}^1D_q[1,1] \\
\hat{a}^{\dagger}_{p,1}\hat{a}_{p,1} & {}^1D_p[1,1]{}^1D_q[0,0] & {}^1D_p[1,1]{}^1D_q[0,1] & {}^1D_p[1,1]{}^1D_q[1,0] & {}^1D_p[1,1]{}^1D_q[1,1]
\end{array}
\tag{11}
$$

${}^1D_i$ is the RDM for qubit i described in Eq. (4) and that ${}^1D_i[a,b]$ is the element of that matrix with matrix coordinates $[a,b]$.

Thus, the overall form of the ${}^2\tilde{G}$ matrix is given by

$$
\begin{array}{|c|c|c|c|}
\hline
p{=}0,q{=}0 & p{=}0,q{=}1 & \cdots & p{=}0,q{=}N{-}1 \\
\hline
p{=}1,q{=}0 & p{=}1,q{=}1 & \cdots & p{=}1,q{=}N{-}1 \\
\hline
\vdots & \vdots & \ddots & \vdots \\
\hline
p{=}N{-}1,q{=}0 & p{=}N{-}1,q{=}1 & \cdots & p{=}N{-}1,q{=}N{-}1 \\
\hline
\end{array}
\tag{12}
$$

where each p/q combination represents the difference of the matrices given in Eqs. (9) and (11), i.e., a block of the ${}^2\tilde{G}$ matrix. The largest eigenvalue of this overall matrix is the λ_G value utilized as the signature of qubit condensation (and hence correlation) throughout this study.

The GHZ states prepared in this study are real wavefunctions, and, hence, the imaginary components of the reduced density matrices are approximately zero within a small range dictated by inherent randomness and by the error of the devices. Thus, only the five two-qubit expectation values corresponding to real contributions to ${}^2\tilde{G}$ [$\langle X_p \otimes X_q \rangle$, $\langle Y_p \otimes Y_q \rangle$, $\langle Z_p \otimes Z_q \rangle$, $\langle X_p \otimes Z_q \rangle$, and $\langle Z_p \otimes X_q \rangle$] are non-zero and thus essential for determination of λ_G. As such, only real components are included in the ${}^2\tilde{G}$ matrix to lower computational expense.

Determination of Shannon entropy.

Shannon entropy (S_e) is determined according to

$$S_e = -\sum_{j=1}^{2^N} p_j \log_2(p_j) \tag{13}$$

where p_j corresponds to the probability of the system to be in the specific j^{th} quantum state of the 2^N possible quantum states for an N-qubit state preparation. These probabilities correspond to the diagonal elements of the full N-qubit density matrix, ${}^2D$. In the case of a two-qubit system, there are 2^2 or four possible states ($|00\rangle$, $|01\rangle$, $|10\rangle$, and $|11\rangle$) with the diagonal elements corresponding to $\hat{a}^\dagger_{0,0}\hat{a}_{0,0}\hat{a}^\dagger_{1,0}\hat{a}_{1,0}$, $\hat{a}^\dagger_{0,0}\hat{a}_{0,0}\hat{a}^\dagger_{1,1}\hat{a}_{1,1}$, $\hat{a}^\dagger_{0,1}\hat{a}_{0,1}\hat{a}^\dagger_{1,0}\hat{a}_{1,0}$, and $\hat{a}^\dagger_{0,1}\hat{a}_{0,1}\hat{a}^\dagger_{1,1}\hat{a}_{1,1}$. These diagonal elements can again be translated into a linear combination of Pauli matrices. For example, $\hat{a}^\dagger_{0,0}\hat{a}_{0,0}\hat{a}^\dagger_{1,1}\hat{a}_{1,1}$ can be written as

$$\begin{aligned}\hat{a}^\dagger_{0,0}\hat{a}_{0,0}\hat{a}^\dagger_{1,1}\hat{a}_{1,1} &= \left(\hat{a}^\dagger_{0,0}\hat{a}_{0,0}\right) \otimes \left(\hat{a}^\dagger_{1,1}\hat{a}_{1,1}\right) \\ &= \left[\frac{1}{2}\left(I_0 + Z_0\right)\right] \otimes \left[\frac{1}{2}\left(I_1 - Z_1\right)\right] \\ &= \frac{1}{4}\left[I_0 \otimes I_1 + Z_0 \otimes I_1 - I_0 \otimes Z_1 - Z_0 \otimes Z_1\right].\end{aligned} \tag{14}$$

The element can then be computed from the appropriate linear combination of the expectation values of Z_0, Z_1, and $Z_0 \otimes Z_1$. This methodology can be generalized to the other elements in the case of a two-qubit quantum system and further generalized to an N-qubit system.

Determination of energy via the QACSE.

To compute the molecular energy of dihydrogen (H_2) with an internuclear distance of 1 Å using the Slater-type orbital with six Gaussians (STO-6G) basis set, we implement the QACSE method—a quantum solver of contracted eigenvalue equations introduced in Ref. 6 with the technique to reduce the number of qubits by tapering being detailed in Ref. 7. The quantum computations were done by using the electron integrals from a full configuration interaction calculation performed via use of the pySCF package. In particular, we perform both the one-qubit and two-qubit calculations under the Jordan Wigner transformation.

Quantum device specifications.

Throughout this work, we employ ibm_lagos, ibm_perth, and ibmq_jakarta IBM Quantum Experience devices, which are available online. These quantum devices are composed of fixed-frequency transmon

qubits with co-planer waveguide resonators [8, 9]. Device specifications are detailed at IBM's Quantum Experience Website [10]. The Python package QISKIT [11] was used to interface with the devices. Each measurement for the calculation of λ_G and S_e were performed with 2^{13} shots, and each measurement for in the QACSE method was performed with 20,000 shots.

* damazz@uchicago.edu

[1] M. Neeley, R. C. Bialczak, M. Lenander, E. Lucero, M. Mariantoni, A. D. O'Connell, D. Sank, H. Wang, M. Weides, J. Wenner, *et al.*, Generation of three-qubit entangled states using superconducting phase qubits, Nature **467**, 570–573 (2010).

[2] L. Dicarlo, M. D. Reed, L. Sun, B. R. Johnson, J. M. Chow, J. M. Gambetta, L. Frunzio, S. M. Girvin, M. H. Devoret, R. J. Schoelkopf, *et al.*, Preparation and measurement of three-qubit entanglement in a superconducting circuit, Nature **467**, 574–578 (2010).

[3] R. Barends, J. Kelly, A. Megrant, A. Veitia, D. Sank, E. Jeffrey, T. C. White, J. Mutus, A. G. Fowler, B. Campbell, *et al.*, Superconducting quantum circuits at the surface code threshold for fault tolerance, Nature **508**, 500–503 (2014).

[4] D. Cruz, R. Fournier, F. Gremion, A. Jeannerot, K. Komagata, T. Tosic, J. Thiesbrummel, C. L. Chan, N. Macris, M.-A. Dupertuis, *et al.*, Efficient quantum algorithms for GHZ and W states, and implementation on the ibm quantum computer, Advanced Quantum Technologies **2**, 1900015 (2019).

[5] M. Treinish and D. M. Rodríguez, GHZ state example, GitHub (2020).

[6] S. E. Smart and D. A. Mazziotti, Quantum solver of contracted eigenvalue equations for scalable molecular simulations on quantum computing devices, Phys. Rev. Lett. **126**, 070504 (2021).

[7] S. E. Smart, J.-N. Boyn, and D. A. Mazziotti, Resolving correlated states of benzyne with an error-mitigated contracted quantum eigensolver, Phys. Rev. A **105**, 022405 (2022).

[8] J. Koch, T. M. Yu, J. Gambetta, A. A. Houck, D. I. Schuster, J. Majer, A. Blais, M. H. Devoret, S. M. Girvin, R. J. Schoelkopf, and et. al., Charge-insensitive qubit design derived from the cooper pair box, Phys. Rev. A **76**, 042319 (2007).

[9] J. M. Chow, A. D. Córcoles, J. M. Gambetta, C. Rigetti, B. R. Johnson, J. A. Smolin, J. R. Rozen, G. A. Keefe, M. B. Rothwell, M. B. Ketchen, and et al., Simple all-microwave entangling gate for fixed-frequency superconducting qubits, Phys. Rev. Lett. **107**, 080502 (2011).

[10] IBM, IBM Quantum Experience (2022).

[11] M. S. Anis, A. Mitchell, H. Abraham, A. Offei, R. Agarwal, G. Agliardi, M. Aharoni, V. Ajith, I. Y. Akhalwaya, G. Aleksandrowicz, *et al.*, Qiskit: An open-source framework for quantum computing (2021).
